# Leishmaniasis: Immune Cells Crosstalk in Macrophage Polarization

**DOI:** 10.3390/tropicalmed8050276

**Published:** 2023-05-15

**Authors:** Fernanda Silva Almeida, Shayenne Eduarda Ramos Vanderley, Fernando Cézar Comberlang, Arthur Gomes de Andrade, Luiz Henrique Agra Cavalcante-Silva, Edson dos Santos Silva, Pedro Henrique de Sousa Palmeira, Ian P. G. do Amaral, Tatjana S. L. Keesen

**Affiliations:** 1 Immunology of Infectious Diseases Laboratory, Department of Cellular and Molecular Biology, Federal University of Paraiba, João Pessoa 58051-900, PB, Brazilshayenne.erv@gmail.com (S.E.R.V.); fcezar14@gmail.com (F.C.C.); arthurg.hit@gmail.com (A.G.d.A.); luiz0710@gmail.com (L.H.A.C.-S.); silvaedson274@gmail.com (E.d.S.S.); phsp@academico.ufpb.br (P.H.d.S.P.); 2 Laboratory of Biochemistry, Department of Cellular and Molecular Biology, Federal University of Paraiba, João Pessoa 58051-900, PB, Brazil; ianamaral@cbiotec.ufpb.br

**Keywords:** leishmaniasis, immune response, macrophage phenotype

## Abstract

Leishmaniasis is a complex infectious parasitic disease caused by protozoa of the genus *Leishmania*, belonging to a group of neglected tropical diseases. It establishes significant global health challenges, particularly in socio-economically disadvantaged regions. Macrophages, as innate immune cells, play a crucial role in initiating the inflammatory response against the pathogens responsible for this disease. Macrophage polarization, the process of differentiating macrophages into pro-inflammatory (M1) or anti-inflammatory (M2) phenotypes, is essential for the immune response in leishmaniasis. The M1 phenotype is associated with resistance to *Leishmania* infection, while the M2 phenotype is predominant in susceptible environments. Notably, various immune cells, including T cells, play a significant role in modulating macrophage polarization by releasing cytokines that influence macrophage maturation and function. Furthermore, other immune cells can also impact macrophage polarization in a T-cell-independent manner. Therefore, this review comprehensively examines macrophage polarization’s role in leishmaniasis and other immune cells’ potential involvement in this intricate process.

## 1. Introduction

Neglected diseases cause major global health problems, predominantly in tropical areas, of which infections caused by protozoan parasites are particularly interesting. Among the protozoa of the Family Trypanosomatidae that can cause human diseases, *Leishmania* is the etiologic agent of leishmaniasis and can cause chronic infection, resulting in several comorbidities [1,2].

Leishmaniasis is endemic in Asia, Africa, the Americas, and the Mediterranean. The transmission occurs through the blood meal carried out by female sandflies. The Americas have about two-thirds of all reported cases, totaling more than 1.3 million new cases annually and an estimated 350 million people at risk of infection. Leishmaniasis is endemic in 17 countries in the Americas, and Brazil is the most affected, with around 96% of all records [3,4,5,6,7,8].

Human leishmaniasis can range from localized cutaneous ulcerative lesions to disseminated mucocutaneous and fatal visceral infections, with four main clinical forms: visceral (LV or calazar), cutaneous or mucocutaneous (LCM), diffuse cutaneous (DCL), and post-kalaazar dermal (PKDL) [9]. In addition, human leishmaniasis is listed by WHO as a priority pathology regarding the development of new treatments [10].

The life cycle of the parasites that cause leishmaniasis is of the heterogeneous type. *Leishmania* has two distinct morphological aspects: promastigote and amastigote. The promastigote stage, the infective form, exists in the fluids of the invertebrate host or the midgut. After being inoculated in the vertebrate host, the promastigotes transform into amastigotes in the mononuclear phagocytes and infect other cells. Once the amastigote stage is established in the vertebrate host, the parasite replicates itself [9,11,12].

Of particular interest to immunologists are the vast nuances in the degree of clinical manifestations and injuries that leishmaniasis cause in infected individuals, which is promoted mainly by the type and amplitude of the host’s immunological response. Pathogenesis results from the interaction between responses developed by the innate immune cells and adaptive immune cells of the infected host [13,14,15,16].

The recruitment of cells and the immediate cellular immune recognition of the parasite begins through the interaction of the parasites with the epithelium, which releases molecular patterns associated with tissue damage. Neutrophils are recruited to the infection site and can influence the outcome of the disease [17]. Quiescent monocytes and macrophages (called M0) are also recruited. After activation, macrophages can display a pro-inflammatory response and are called M1 macrophages. Alternatively activated macrophages develop functions to control the exacerbated inflammatory process and tissue repair, in which case they are classified as M2 macrophages [18,19,20].

The interaction between lymphocytes [21] and macrophages [22] in leishmaniasis has a bidirectional role in activation and metabolism. The Th1 lymphocyte profile induces differentiation of M0 macrophages into M1 through the secretion of pro-inflammatory cytokines such as interferon-γ (IFN-γ) and tumor necrosis factor-alpha (TNF-α). Mitochondrial metabolism and the generation of reactive oxygen species (ROS) are increased in M1 macrophages, resulting in damage and death of the parasites *Leishmania* in the infectious process [20,23].

However, Th2 lymphocytes released IL-4 and IL-13 and thus induced the differentiation of M0 macrophages into M2 (alternative activation via). The metabolism of M2 macrophages creates an environment with essential metabolites which help the maintenance and parasite survival [20,23].

A successful cellular immune response requires interactions with several cellular subgroups and a cytokine collaborative network [24,25]. Thus, this work reviewed macrophage polarization and how other immune cells could be involved in the dynamics of macrophage plasticity, as well as their role in Leishmaniasis infections.

## 2. Macrophages M1/M2 Polarization

Macrophages, as key players in developing innate responses, are cells whose intrinsic characteristic is the ability to be plastic in response to numerous stimuli. This plasticity refers to the polarization of these cells, by which macrophages can differentiate into different cell phenotypes. This results in macrophages with various physiological functions, such as the production of cytokines, phagocytosis, tissue repair, the proliferation of stem cells, angiogenesis, and fibrosis [26,27,28].

The polarization capacity of macrophages results from broad pathways via epigenetics and the tissue microenvironment since they are cells that are highly resident in the tissues. Also, via extrinsic factors triggered by antigenic stimuli or cytokines. Most macrophages residing in the tissue are from a hematopoietic parent common to monocytes. The maturation of these macrophages will occur depending on specific endogenous and exogenous stimuli [29,30].

The process of macrophage polarization is an essential target for studies of how these cells behave in inflammatory environments generated by infectious processes, such as leishmaniasis. In addition, several studies have pointed out macrophages as a critical element in innate and adaptive immune response, having a regulatory role in the inflammatory process [31].

**Figure 1:**
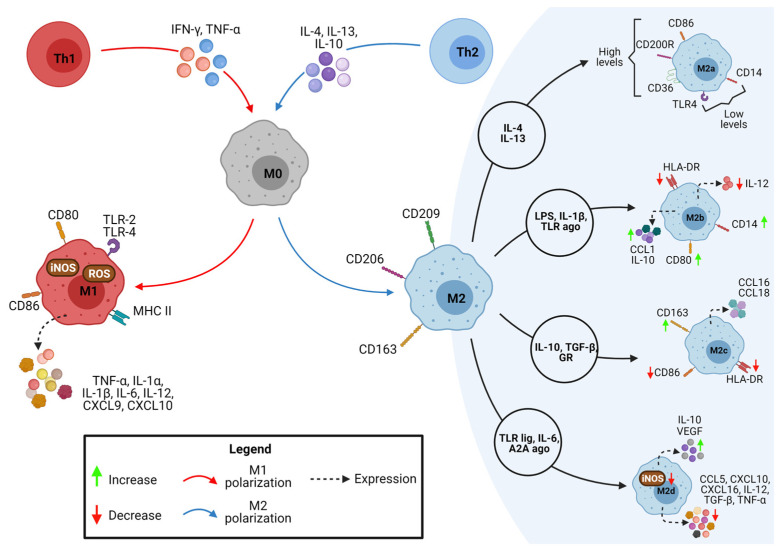
Classically and alternatively activated macrophages. Macrophages represent an essential innate immune cell component characterized by their plasticity. When exposed to specific microenvironmental conditions, macrophages can suffer polarization, originating distinct phenotypes. For instance, cytokines such as IFN-γ and TNF-α (also expressed by Th1 cells) can lead to the polarization of M0 macrophages into pro-inflammatory profiles called classically activated macrophages (M1). These subpopulations are characterized by the expression of surface markers (TLR-2, TLR-4, CD80, CD86, MHC-II) and secretion of cytokines and chemokines such as TNF-α, IL-1β, IL-6, CXCL9, and CXCL10. On the other hand, alternatively activated macrophages (M2) can be generated through interaction with Th2 cytokine profiles like IL-4, IL-13, and IL-10 and can express CD206, CD163, and CD209. Noteworthy, based on the stimulus that these cells are exposed to, M2 macrophages can be divided into four subtypes with distinct phenotypes: M2a (generated by interaction with IL-4 and IL-13), M2b (activated in response to lipopolysaccharide (LPS), IL-1β, TLR agonists and some immune complexes), M2c (via IL-10, TGF-β, and glucocorticoids) and M2d (activated by IL-6, TLR, and A2A adenosine receptor agonists). Created with BioRender.com.

The quiescent macrophages (M0) can adopt different phenotypes when exposed to factors that promote the polarization process. For example, classically activated M1 macrophages establish a pro-inflammatory profile known as CAM (classically activated “inflammatory” macrophages) phenotype. After activation of M0 macrophages with cytokines such as IFN-γ and TNF-α, macrophages acquire the M1 phenotype, characterized by the expression of surface markers, such as TLR-2, TLR-4, CD80, CD86, MHC-II, and iNOS. In addition, M1 macrophages secrete cytokines such as TNF-α, IL-1α, IL-1β, IL-6, IL-12, CXCL9, and CXCL10 (Figure 1) [26,27,32,33,34].

The alternative pathway of macrophage activation develops a regulatory profile (also called remodeling or anti-inflammatory profile) or M2 macrophages with AAM phenotype (alternatively activated “reparative” macrophages). M0 is activated in M2 through interaction with IL-4, IL-13, and IL-10 cytokines. These cells express the surface markers CD206, CD163, and CD209 (Figure 1). The plasticity of the macrophage polarization profiles is linked to the characteristics of the environment. Therefore, changes in the microenvironmental dynamics can modulate the phenotype of macrophage subpopulations. Polarization for the M2 profile is also considered a pro-resolving response associated with subsequent infection stages and control of inflammation [26,27,31,32,33,34].

Innate and adaptive immunity are concurrently orchestrated responses to control the invasive agent, promoting the clearance of infection, and resuming homeostatic balance, through cellular communication facilitated by molecules produced by the various cell subtypes [35]. For example, in the case of macrophages, the control of the polarization plasticity of the M1 and M2 macrophages can be caused by cytokines produced respectively by the CD4^+^ Th1 and Th2 lymphocyte subpopulations [31].

Alternatively activated macrophages, or M2, can be divided into 4 cell subtypes (i.e., M2a, M2b, M2c, and M2d) based on the stimuli they are exposed to. M2a activation is developed in response to interaction with IL-4 and IL-13, showing high levels of CD86, CD200R, and CD36 expression, with CD36 being a crucial hijacker receptor in resolving the inflammatory process. M2a macrophages also have low levels of CD14 expression and TLR4. M2b is activated in response to stimulation with lipopolysaccharide (LPS), IL-1β, TLR agonists, and immune complexes, the latter being recognized by the family of Fcγ receptors (CD64, CD32, CD16a, and CD16b). Activation of the M2b profile results in increased expression of CD80, CD14, CCL1, and IL-10, production of cytokines with pro-inflammatory characteristics, reduced secretion of IL-12, and expression of human leukocyte antigen (HLA-DR). Interaction with glucocorticoids, IL-10, and TGF-β causes M2c activation, leading to increased expression of CD163 and decreased expression of CD86 and HLA-DR, in addition to the production of CCL16 and CCL18 [20,31]. M2d is a phenotype activated via IL-6 interaction, Toll-like receptor ligands (TLR), and agonists of the A2A adenosine receptor and are known as tumor-associated macrophages (TAMs). M2d macrophages have high expression of anti-inflammatory and angiogenic factors, such as IL-10 and VEGF, in response to adenosine receptor agonists. In addition, M2d macrophages secret chemokines such as CXCL10, CXCL16, and CCL5 and have low production of IL-12, TGF-β, iNOS, and TNF-α (Figure 1) [20,31,32,36].

Therefore, macrophage activation and polarization correlate with cell proliferation events and/or the development of effector functions that allow these cells to integrate and act on immune responses. In addition, macrophages can be activated and stimulated to produce cytokines and chemokines that can influence other cells’ biology. For example, macrophages can promote the effector response of other immune cells. Thus, activation and polarization may be affected by the direct exposure to signals broadcast by a pathogen or an ongoing infection and indirectly by the cytokines produced by other cells, whether immune or not immune [37].

The metabolic characteristics and transcription factors [38] of macrophages regulate their functions, including the release of cytokines and the expression of the cell surface receptor. M1 and M2 macrophages are examples of this concept. The main metabolic and functional differences between the polarization states of the M1 and M2 macrophages in vitro is that the M1 macrophages, by stimulating the TNF-α or IFN-γ, increase glycolytic metabolism to generate ATP. This favors phagocytic and microbicide functions while feeding an interrupted mitochondrial tri-carboxylic acid (TCA) cycle. Thus, M1 macrophages produce inflammatory cytokines and reactive oxygen species, essential for the host’s defense against infections and the early response to tissue damage [33,39]. However, M2 macrophages release anti-inflammatory cytokines and may be related to promoting angiogenesis and fibrosis. In addition, M2 macrophages use fatty acid metabolism and oxidative mitochondrial phosphorylation (OXPHOS) and mediate host responses to parasites [33,40,41].

The metabolic phenotype of classically activated macrophages (M1) is characterized by high rates of anaerobic glycolysis even in the presence of oxygen, a metabolic characteristic known as Warburg metabolism [42,43]. In addition, M1 macrophages increase glucose uptake due to increased transcription and translocation of the glucose transporter 1 (GLUT1) [44]. In Warburg’s metabolism, the TCA cycle in pro-inflammatory macrophages (M1) is incomplete, and citrate and succinate are accumulated [30,44,45].

Pro-inflammatory macrophages are characterized by slower mitochondrial phosphorylation (OXPHOS), even with increased glycolytic rate and decreased ATP production through the electron transport chain (ETC). The modification of the electron transport chain and inhibition of mitochondrial respiration is modulated by nitric oxide (NO). Thus, M1 macrophages exhibit increased glycolysis, complete suppression of OXPHOS, and reduced fatty acid oxidation (FAO) [30,33,46,47,48].

In M2 macrophages, the main metabolic signature is the consumption of fatty acid and increased mitochondrial respiratory capacity. M2 macrophages are also frequently associated with a metabolism other than arginine. For example, the induction of nitric oxide synthase 2 (NOS2) in M1 macrophages metabolizes arginine to produce NO. However, in the alternatively activated macrophages, the activity of the enzyme arginase, which alternatively processes arginine into ornithine, has proline and polyamines [30,49,50].

Alternatively activated macrophages (M2) have elongated mitochondria as a characteristic and, consequently, more efficient energy production. These cells use fatty acids, glucose, and glutamine to feed the TCA cycle and produce ATP by oxidative phosphorylation. CD36 absorbs exogenous fatty acids and triacylglycerols, and lipolysis generates free fatty acids for fatty acid oxidation (FAO). Activated fatty acids enter the mitochondria, which create acetyl-CoA, to contribute to the TCA cycle. In addition, acetyl-CoA is used in the acetylation of histones associated with genes that are targets of IL-4 signaling [37].

In a study by Ty et al. (2019), the metabolic and immunological status of human macrophages was investigated after infection by *Leishmania donovani* and *L. amazonensis* and their ability to respond to a classic polarizing stimulus (LPS and IFN-γ). Infection of *Leishmania* macrophages resulted in the activation of oxidative phosphorylation. Furthermore, infected cells still respond to activation by adding stimuli (LPS + IFN-γ) to *L. donovani*-infected macrophages, which promotes a significant increase in glycolysis. In addition, there was an increase in inflammatory cytokine secretion compared to uninfected macrophages, indicating that infected macrophages increased metabolic capacity with increased cytokine production in response to stimuli [51]. In an experimental model of *Leishmania* infection using THP-1 cells, significant changes in these cells’ metabolic profile were reported, promoting the ongoing infectious process since *Leishmania* within the parasitophorous vacuole, *Leishmania* can sequester nutrients from cellular metabolism and increase their survival through host cellular machinery [52].

Ty et al. (2019), in their assay on macrophages derived from human monocytes, found no significant increase in glycolysis or oxidative phosphorylation. Furthermore, in this assay, there was no induction of inflammatory cytokine secretion by infected macrophages, typical of the M1 phenotype. However, activation with IFN-γ and LPS in infected macrophages can induce glycolysis and inflammatory cytokines, indicating that the parasite infection did not inhibit M1 polarization. This may suggest that, during infection, macrophages are still able to respond effectively to an external activation stimulus. Thus, preventing macrophage cytokine responses help parasites to establish a persistent infection, opening new possibilities for further study as a therapeutic strategy [51].

## 3. M1 and M2 Macrophages in Cutaneous Leishmaniasis

It is generally considered that the success of the infection process in host macrophages by *Leishmania* is the result of an inadequate or incomplete immune response. Phagocytic cells such as neutrophils, dermal dendritic cells, and dermal macrophages are the preceding line of defense that recognize infection by the promastigote form of *Leishmania*, resulting in an innate immune response of a pro-inflammatory character [53]. In addition to having the ability to produce antileishmanial effector molecules, these cells also comprise the refuge niche for the parasite to escape the humoral cytotoxic components [54,55,56].

In experimental models infected with *L. major*, BNI isolated, using normally resistant B6 wild mice and mice with TNF B6 deficiency (B6.TNF^−/−^), progression to a severe or lethal infection profile was reported in the absence of TNF. The infection reached a systemic level on the 35th day, with a significant increase in the size of visceral organs, such as the liver. In addition, a substantial increase in cytokines with pro-inflammatory characteristics MCP-1, IL-6, and IFN-γ has been reported in B6. TNF^−/−^ mice. The profile of severe infection was correlated with the decreased polarization to the M1 phenotype due to TNF deficiency and increased M2 phenotype. Thus, the authors concluded that M1 macrophages control the progression and installation of *L. major* BNI infection [57,58].

The interaction between innate and adaptive immunity in the context of intracellular infections is one of the crucial factors in the classic versus alternative activation pathways, leading to resistance or susceptibility to parasite infection. The role of cytokines that induce macrophage polarization is widely studied. Using human recombinant cytokines such as IL-15 and IL-32, a study showed their role in controlling infection by *L. braziliensis*. IL-15 cytokine is an activator of the Th1 profile and NK cells. Activation of the Th1 profile results in polarization of M0 into M1 macrophages, whereas activation of NK cells results in higher production of IFN-γ. Like IL-15, IL-32 can stimulate NK cells and induce the production of IFN-γ. Furthermore, macrophage exposure to IL-15 and IL-32, besides favoring polarization for the M1 phenotype, increases microbicidal activity by increasing reactive oxygen and nitrogen species. Thus, both cytokines work in synergy to resolve infection in human macrophages [50,59].

**Figure 2:**
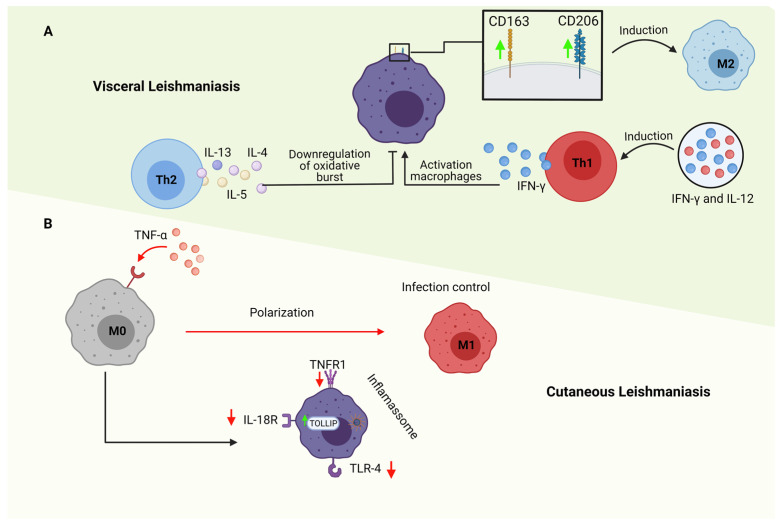
Immune modulation of macrophages during leishmaniasis. (**A**). Visceral Leishmaniasis has higher serum levels of pro-inflammatory cytokines IFN-γ and IL-12 and immunosuppressors IL-10. IL-12 can be expressed by infected dendritic cells, and such as INF-γ can drive to Th1 response. On the other hand, cytokines characteristics of the Th2 subtype, like IL-4, IL-5, and IL-13, lead to the growth of the parasite and help in the persistence of infection in chronic diseases. They also negatively modulate the oxidative burst in macrophages. Moreover, CD206 and CD163, markers that favor M2 polarization, had increased expression in macrophages infected with *Leishmania donovani*. (**B**). TNF-α is an essential cytokine in promoting M1 polarization. In animal models, the deficiency of these proinflammatory molecules during *Leishmania major* infection leads to a severe disease progression with a decrease in M1 macrophages and enhancement in M2 phenotype formation, suggesting the rule of these inflammatory profiles in control of infection. Moreover, *Leishmania amazonensis* can modulate NRLP3 inflammasome complexes and reduce positive NF-kB regulators like IL18R1, TNFRSF1A, Toll-like receptor 4 (TLR4) and MYD88 and increase anti-inflammatory molecules as TOLLIP, an inhibitor of TLR. Created with BioRender “https://app.biorender.com/ (accessed 21 April 2023)”.

Immune escape pathways by the various pathological agents are widely and extensively studied. As the immune responses evolve to a better response to secondary infection, the pathogens also evolve to evade immune control. Assays using epigenetic regulation revealed that *L. amazonensis* infection could significantly modulate some inflammasome components in macrophages, such as NLRP3, NLRC4, AIM2, and RIG-1. Furthermore, it is known that the NRLP3 inflammasome pathway activates caspase 1 that, cleaves the pro-IL-1b and pro-IL-18 factors in the cell cytoplasm, thereby secreting IL-1b and IL-18, whose functions in this context are pro-inflammatory. In addition to the NRLP3 inflammasome pathway, *L. amazonensis* reduced the expression of positive regulators of the NF-kB pathway, such as the IL18R1, TNFRSF1A, TLR4, and MYD88 surface pro-inflammatory receptors, and positively regulated anti-inflammatory molecules and inhibitors known as TOLLIP, an inhibitor of TOLL type receptors [60] (Figure 2).

## 4. M1 and M2 Macrophages in Visceral Leishmaniasis

Macrophages are the central host-capable cell for *Leishmania* spp., and the secretion of macrophage-derived cytokines, such as TNF-α and IL-12, modulate the treatment of leishmaniasis [61]. However, other innate cells also contribute to resistance and/or susceptibility to *Leishmania* infection [62]. According to Dos Santos et al. (2016), it was observed that patients with VL have high levels of IFN-γ and IL-12 in the serum, which could be linked to help in controlling the course of infection. However, an increase in anti-inflammatory mediators has also been reported, for example, IL-10, which can negatively interfere in the fight against the parasite and enable its multiplication, thus inducing the worsening of the infection [63].

Many genes, including the mannose receptor (CD206), arginase 1 (ARG1), heme receptor (CD163), and inducible nitric oxide synthase (iNOS), are involved in regulating the polarization of M1 and M2 macrophages [64,65] (Figure 2). Macrophages residing in the liver (i.e., Kupffer cells—KCs), reservoirs of the intracellular amastigote form in visceral leishmaniasis, constitute about 80 to 90% of the macrophage population throughout the body and are characterized by the expression of characteristic markers of macrophages (F4/80, CD14, CD68, CD11b), as well as by lectin type C (Clec)-4F. In addition, KCs maintain a characteristic anti-inflammatory environment through various effector mechanisms, such as IL-10 secretion, diminished MHC-II expression, and elevated PDL-1 expression. These mechanisms limit the ability to present antigens, thus exhibiting characteristics of the macrophage profile M2 [64].

The infected dendritic cells in resistant animals produce IL-12, a cytokine that favors Th1 responses, establishing a characteristic pro-inflammatory environment. The cytokine IL-12 also promotes responses from CD4^+^ T cells that act on NK and NK T cells, providing positive feedback, with the production of IFN-γ further polarizing Th1 responses [66]. As a way of attenuating the pro-inflammatory response, the pathway involved in regulating the cellular inflammatory response results in the promotion of the infection process. This is the case in which IL-4 produced by mast cells and other innate cells at the initial infection site leads to the induction of responses characterized as anti-inflammatory Th2 polarization. The cytokines produced by the anti-inflammatory CD4^+^ Th2 cells, which include IL-4, IL-5, and IL-13, lead to the growth of the parasite and the persistence of infection in chronic diseases, in addition to playing a role in the negative regulation of the oxidative burst in infected macrophages [67,68,69].

The balance between the pro-inflammatory and anti-inflammatory cytokines influences the infection’s outcome regarding the pathophysiology progression, where its polarization, resistance, or susceptibility effects are not fully elucidated in human VL [69]. In a complementary way, the pathogenesis of visceral leishmaniasis is correlated with high serum levels of the immunosuppressive cytokine IL-10, which makes it possible to promote the growth of *L. donovani* amastigotes in human macrophages. These examples indicate that even though the host’s immune responses play a determining role in modulating the control of *Leishmania* infection, the interactions of the host’s immune response to the ongoing infection are also significant regarding the progression of pathophysiology [69].

In dogs, the main effector mechanism triggered by the protective immune response is the release of cytokines such as IFN-γ and TNF-α, which promote the activation of macrophages, inducing the death of intracellular amastigotes. This protective immune response provides apparent resistance to visceral leishmaniasis, preventing the evolution of the pathology. However, *Leishmania* produces a series of factors that reduce the microbicidal mechanisms of macrophages, thus evading the immune response. The attenuation of the macrophage immune response depends on an increase in the Th2 response and the secretion of IL-4 and IL-10. Although the role of these cytokines in symptomatic animals is still debatable, there is growing evidence of a correlation between these cytokines and the evolution of the disease [70].

According to Moreira et al. (2016), the spleen of *Leishmania*-infected dogs presents a high parasite burden, suggesting that it would be more susceptible to the multiplication of this parasite. On the other hand, the liver seems to be a less favorable environment for the parasite in these animals due to its low parasitism. Furthermore, the polarization of macrophages to the M2 phenotype may benefit the survival of *Leishmania*, creating an anti-inflammatory microenvironment that prevents a Th1 immune response against *Leishmania* [70].

Kong et al. (2017), seeking to understand the dynamics between the tissue affected by the infection and the role of macrophages in the immunopathogenesis of Leishmaniasis, have investigated gene expression in infected spleens and splenic hamster macrophages by RNA sequencing (RNA-Seq). Based on the transcriptional profile of the spleen, hamsters infected with *L. donovani* proved to be carriers of a surprisingly pro-inflammatory environment. The expression of transcriptions factors genes that drive inflammation (e.g., STA3) and MHC expression was upregulated. In addition, the transcription factors involved in the dynamics of the inflammatory response, such as the NF-kB, CBP/P300, and DDIT3 complex (activation of caspase, expression of cytokines), were activated in the infected spleen [71]. Contrary to what was expected, M1 polarization was not essential for parasite clearance, and IFN-γ spontaneously increased parasite growth. A suggestion for this scenario would be that as the VL progresses, the splenic macrophages in VL are conditioned by the chronic inflammatory environment to respond convergently to the signs of macrophage activation in a pathological and aberrant manner to favor an infection [71].

## 5. Leishmaniasis: Immune Cells Crosstalk in Macrophage Polarization

Through different mechanisms, immune cells communicate with each other during macrophage polarization [27]. This crosstalk helps determine the functional phenotype of macrophages and their role in the immune response. In the following sections, we described the possible role of innate and adaptative cells in macrophage polarization and their correlation with leishmaniasis.

### 5.1. ILCs

Innate Lymphoid Cells (ILCs) are a recently discovered family of immune cells that play a crucial role in maintaining tissue homeostasis, promoting immunity, and mediating tissue repair. These cells differ from adaptive lymphocytes as they lack rearranged antigen receptors that recognize foreign structures. However, they exhibit a similar functional diversity to T cells, despite being unable to identify ‘non-self’ antigens through antigen receptors. ILCs are classified into three main groups based on their transcription factor and cytokine expression patterns: ILC1, ILC2, and ILC3 [72].

ILC1s resemble Th1 cells and produce IFN-γ, indispensable for host immunity to intracellular parasites (e.g., *Leishmania* spp.). ILC2s produce type 2 cytokines, including IL-4, IL-5, and IL-13, and play a critical role in allergic and helminth infections. Finally, ILC3s produce IL-17 and IL-22, which are involved in the defense against extracellular pathogens and tissue repair [73].

ILCs act as sentinel cells, initiating rapid changes in tissue responses to restore homeostasis or alert the immune system as needed, recruiting various leukocytes. In addition, some studies have suggested that ILCs may be crucial in the initial immune response to infections [74]. Therefore, the interaction between macrophages and ILCs may be the critical factor in promoting the early feedforward process of the immune response in infectious diseases, such as *Leishmaniasis*.

A study by Rodríguez et al. (2021) showed the variability of different proportions of ILC phenotypes in other forms of Leishmaniasis. For example, LCL (Localized Cutaneous Leishmaniasis) has a higher presence of ILC1 and ILC3 responses. Therefore, it may be responsible for promoting a Th1 response, which can help control the infection [75]. Specifically, in LCL patients, a unique subtype of ILC1 that requires T-bet for differentiation can produce significant amounts of IFN-γ and TNF-α when exposed to cytokines from infected cells, resulting in a more robust Th1 response and consequent classical macrophage activation, as previously observed in inflammatory bowel disease (IBD) and infection-induced colitis [76,77]. Besides, when exposed to pro-inflammatory cytokines produced by ILC1, the ILC3 subtype can generate GM-CSF, which plays a crucial role in M1 macrophage polarization [78]. This process is essential for the body’s protective response against *Leishmania* infections. On the other hand, in patients with DCL (Diffuse Cutaneous Leishmaniasis), there is a higher incidence of ILC2 and ILC3, suggesting an inclination to M2 macrophage phenotype, which results in type 1 response impairment, as observed in patients presenting T cell anergy and disseminated disease [75].

Despite the ILC2 predominance, in mouse skin lesions caused by *L. major* infection, it has been shown that eosinophils, rather than ILC2 cells, were the main source of IL-4 during the early stages of the disease [79]. IL-4 is a crucial factor in the expansion and differentiation of Th2 cells, which are critical for inducing alternative macrophage polarization. This type 2 response is characterized by the impaired killing of *Leishmania* parasites, leading to the progression of the disease [80].

Not only do the infection repercussions impact ILCs responses, but the microbiota seems to induce specific responses leading to skin injuries and even play a role in *L. major* infection resistance [81]. The ability to significantly alter the skin microbiota of humans and mice, leading to dysbiosis, has been demonstrated, characterized by a dominance of *Staphylococcus* and/or *Streptococcus*. Thus, acquiring a dysbiotic microbiota before infection can significantly exacerbate skin inflammation in response to *Leishmania* infection. This suggests that dysbiosis may result from infection and contribute to disease pathogenesis [82]. RORγt+ IL-17A-producing ILC3 is involved in microbiota-driven immunopathology. A study by Singh et al. (2021) showed that these cells were enriched in *L. major* infection and colonized skin with *Staphylococcus epidermidis*, leading to augmented skin inflammation in cutaneous leishmaniasis, without affecting type 1 immune responses [83], which is also observed in the generation of IL-17-producing T cells [84].

IL-17A signaling induces macrophage activation in a unique manner that differs from other T cell-derived cytokines but also has an essential role in promoting skin inflammation alongside tissue resident nonimmune cells [85,86]. Furthermore, even without T cells, ILCs and NK cells appear involved in an IL-17-mediated neutrophil accumulation and classical macrophage activation in cutaneous leishmaniasis [87].

Group 1 innate lymphoid cells (ILCs) consist of ILC1s and natural killer (NK) cells. NK cells comprise 5–20% of peripheral blood mononuclear cells (PBMCs) in humans and play an important role in pathogen infection through their cytotoxic effects and pro-inflammatory activities without the need for prior sensitization [88]. NK cells are identified as CD3^−^CD56^+^ in humans [62] and, according to Bellora et al. (2010) [89], are significant producers of T helper cytokines (Th1), such as IFN-γ. In addition, these cells can activate by IL-12, IL-15, and IL-18 and by the interaction between NK activating receptors (NKp46, NKp30, NKp44, DNAM-1, and NKG2D) and their ligands on target cells [89].

Recognition of *Leishmania* LPG via TLR-2 activates NK cells and induces the production of IFN-γ, TNF-α, and translocation of NF-kB to the nucleus. Furthermore, it has been observed that the cytotoxicity and IFN-γ production by this subpopulation also depends on antigen recognition via TLR-9 [62]. However, divergences in the role of NK cells in different *Leishmania* parasites are reported [62], and this is because NK cells still need to be fully elucidated in the context of *Leishmania* infection [88]. Furthermore, these cells are essential for eliminating *L. donovani* amastigote forms but are not necessary for establishing an effective Th1 response against *L. major* and *L. tropica* [62].

The differentiation state of NK cells modulates their functional capacity. It can be divided into phenotypic and functional subsets based on the active expression ratio of CD56 and CD16 on the cell surface. The CD56^bright^ NK subset increases its immunoregulatory and proliferative capacity after cytokine stimulation, while the CD56^dim^ cells, representing the significant subset (~90%), are the most differentiated [62].

The role of NK cells in CL has been associated with pathology and protection. A protective function has been proposed through the lysis of extracellular promastigotes and infected macrophages and a contribution to exacerbating tissue damage by cytotoxic NK cells [90]. It has been shown that NK cells present an exhaustion profile mediated by *Leishmania* antigenic stimulus in CL patients before, during, and after antimonial therapy when cultured with or without *L. braziliensis* antigens. Additionally, in this same study, the expansion of NK cells activated by cytotoxicity was observed before and during treatment, indicating specificity in the response of these cells against *L. braziliensis* [91].

Only 5% of NK cells express CD107a (degranulation profile), demonstrating a weak involvement of the NK cell population in cytotoxicity. We also observed a low frequency of cytotoxic NK cells in the lesions (8% of all CD107a^+^-cytotoxic cells), suggesting that these cells have little influence on the cytotoxicity that occurs in the lesion environment, based on the distribution of total cytotoxic cells in CL caused by *L. braziliensis* [90].

Covre et al. (2020) observed the accumulation of circulating NK cells with multiple replicative senescence characteristics, including low proliferative capacity and shorter telomeres, elevated expression of CD57 and KLRG1, but decreased expression of the CD27 stimulatory receptor, as well as higher cytotoxic and inflammatory potential compared to control groups [88]. In addition, the accumulation of circulating senescent NK cells (CD56^dim^ CD57^bright^) positively correlated with the size of the cutaneous lesion. This profile was also observed in senescent NK cells in the skin, albeit with less evidence proportionally. On the other hand, patients with visceral leishmaniasis present three different subsets of NK cells: CD56^−^CD161^+^, CD56^+^CD161^−^, and CD56^+^CD161^+^, as well as loss of the CD56^+^CD161^+^ subset compared to healthy individuals [62].

Findings demonstrate that interacting unpolarized or polarized NK cells and macrophages result in different functional outcomes [89]. In human and mouse models, it has been observed that co-culturing *Leishmania*-stimulated monocytes or macrophages with NK cells results in positive regulation of CD69 on the surface of NK cells, production of IFN-γ, and degranulation of these cells [92].

In the context of polarization, it is understood that activated NK cells can lyse M0 and M2 macrophages, while M1 macrophages are resistant to lysis [93]. This occurs after stimulation by microbial products such as LPS and Bacillus Calmette-Guérin, where M0 and M2 macrophages polarize towards M1 and induce strong activation of resting NK cells, resulting in increased cytolytic activity, the release of large amounts of IFN-γ, and expression of CCR7, a chemokine receptor crucial for their recruitment to lymph nodes. In turn, activated NK cells kill polarized M0 and M2 macrophages, which express low and non-protective amounts of HLA class I molecules. On the other hand, M1-polarized macrophages (with high HLA class I), like mDCs, are resistant to NK cells [89].

However, the role of NK cells in the polarization and depolarization of macrophages is still uncertain, as there is a gap in the literature on the subject, not only in vitro and in vivo studies and clinical forms of leishmaniasis but also in other disease models, which makes it challenging to make definitive statements on this topic. Therefore, research on this topic is necessary.

Despite recent advances in understanding the role of ILCs in immunity, there is still much to elucidate about how these cells respond to specific pathogens and how that influences macrophage polarization in *Leishmania* infection.

### 5.2. NKT

The NKT cells are a subset of T cells that recognize glycolipid antigens presented through CD1d by APCs [94]. Additionally, like NK cells, they exhibit cytotoxic activity upon TCR binding or after IL-2 production by cells [95]. NKT cells are characterized by the expression of CD4 or CD8 and the production of IFN-γ, TNF-α, IL-4, 10, and 13. They represent 0.1–0.5% of peripheral blood leukocytes and participate in various diseases, including leishmaniasis [94,95,96].

Currently, NKT cells are classified into two subsets: Type 1 NKT cells (iNKT) that express semi-invariant TCRs reactive to CD1d and endogenous and exogenous lipid antigens, and Type 2 NKT cells, which are also restricted to CD1d but do not express the invariant Vα14-Jα18 TCR chain [94]. Type 2 NKT cells present diverse TCRα and β chains and recognize sulfatide or lysophosphatidylcholine (LPC) antigens [96]. iNKT cells represent 70% of NKT cells and are the most well-described. iNKT cells recognize the glycolipid α-galactosylceramide (α-GalCer), while type 2 NKT cells are less frequent and do not recognize α-GalCer [95].

NKT cells play an essential role in leishmaniasis because they exhibit cytotoxicity against cells that cannot be lysed by NK cells through recognition of CD1d, such as *L. infantum*-infected dendritic cells that are protected from NK cell-mediated cytolysis by increased expression of HLA-E during infection [94].

In the early stages of visceral leishmaniasis (VL), NKT cells protect against the disease [62]. CD8^+^ NKT cells are protective, express IFN-γ and Killer immunoglobulin-like receptors (KIRs), and do not migrate to the site of *L. donovani* infection [97]. On the other hand, CD4^+^ NKT cells are considered pathogenic because they migrate to the area of the disease and express CD25, FoxP3, and IL-10. However, it has been shown that the CD3^+^CD56^+^ subset of NKT cells independent of CD1d has a regulatory function by contributing to IL-10 and FoxP3 [98]. In contrast, CD3^+^CD4^+^CD56^+^ NKT cells have a pathogenic profile because they accumulate at the site of infection and down-regulate CD3^+^CD8^+^CD56^+^ NKT cells during visceral leishmaniasis, which is due to the higher expression of CCR5 by CD4^+^CD56^+^ NKT cells compared to CD8^+^ NKT cells [98].

In cutaneous leishmaniasis (CL), NKT cells were the fourth most prevalent population in studies [90] that observed CD107+ degranulating cells with high cytotoxic activity in lesions caused by *L*. *(Viannia) braziliensis*. Among the evaluated subpopulations, NKT cells were the second population with the most increased cytotoxic and degranulating activity (25.0 ± 4.1%). Similarly, it was observed that CD3+CD56+CD8+ NKT cells were associated with a cytotoxic response against infection by *L. (Viannia) braziliensis* [98]. A study suggests that CD8^+^ NKT cells are the main subset involved in cytotoxicity and offer a protective role for the CD4^+^CD8^+^ NKT subset in CL, although studies of this subset in CL are still considered scarce [91].

A study by Gois et al. (2018) suggests that iNKT cells may exhibit plasticity and be involved in distinct mechanisms in the active clinical forms of leishmaniasis via whole blood [99]. In CL, they trigger activation and a pro-inflammatory profile in early *L. braziliensis* infection characterized by an increase in CD69, IFN-γ, and IL-17 expression (in response to *Leishmania* antigen stimulus), and in VL, an initial immune response impaired to *L. infantum* infection characterized by reduced IFN-γ in response to *Leishmania* antigen stimulus and no alteration of other markers when compared to control groups.

Two mechanisms can activate NKT cells: (a) the direct pathway after binding their invariant TCR to CD1d loaded with a glycolipid and (b) the indirect pathway through cytokines such as IL-12 or IL-18 produced by APCs. Both mechanisms can observe this activation of NKT cells in leishmaniasis. The direct pathway activation of NKT cells occurs when *Leishmania* glycocalyx antigens are presented by CD1d and bind to iNKT cells due to their similarity to a-GalCer. In addition, it has been observed that LPG can activate NKT cells from *L. donovani* when bound to CD1d through isoelectric focusing. This activation leads to the subsequent production of cytokines IFN-γ and IL-4 [95].

In contrast, it has been demonstrated the activation of these cells through the indirect pathway, where LPG from *L. mexicana* activates dendritic cells (DCs) through TLR2, leading to the release of IL-12p70 and increased expression of co-stimulatory molecules CD86 and CD40 in DCs, which in turn induces polarization in the production of IFN-γ by NKT cells. According to Cruz et al. (2022), evidence indicates that the crosstalk between NKT cells and macrophages mainly depends on antigen presentation by CD1d, in some cases, on innate mechanisms that are not yet well understood [100]. A study from Beattie et al. (2010) has shown increased activation of iNKT cells but with low levels of IFN-γ production in the presence of *L. donovani*-infected Kupffer cells [101].

However, little is known about whether or how NKT cells are responsible for polarizing and depolarizing macrophages during leishmaniasis. Therefore, NKT cells may polarize macrophages during leishmaniasis based on the direct activation mechanism of these cells but in other disease models. For example, a study demonstrated that NKT cells activated by α-GalCer increase the expression of M1 macrophages iNOS^+^ and Th1 effector cells while reducing the frequency of M2 macrophages CD206^+^ in the tumor microenvironment [93].

Another study by Grabarz et al. (2018) [102] observed a reduction in the activity of M2 macrophages and a decrease in the molecular expression of arginase-1 when type 1 and 2 NKT agonists (α-GalCer and sulfatide) were administered in fibrotic lung tissue. However, a possible mechanism for macrophage polarization via cytokine-activated NKT cells (i.e., indirect pathway) in leishmaniasis cannot be suggested due to the lack of studies addressing the topic. Therefore, additional research is necessary to clarify the role of NKT cells and their activation mechanisms in macrophage polarization during different clinical forms of the disease.

### 5.3. Neutrophils

Neutrophils, polymorphonuclear leukocytes, are highly abundant in human blood and possess significant migratory capacity into tissues. These cells are crucial in the body’s response to infection and inflammation, release antimicrobial substances, and regulate inflammation [103]. In addition, neutrophils interact with other immune cells, such as macrophages, to orchestrate a coordinated immune response [104,105,106].

Macrophage polarization can be influenced directly by neutrophils. During an immune response, macrophages can release chemokines (e.g., CXCL-1) involved in neutrophil recruitment, and soon these cells become apoptotic after effector mechanisms, M1 macrophage initiate phagocytosis process. This event led macrophages to acquire an M2 phenotype to restore homeostasis [107]. Also, in helminth infections, Chen et al. (2014) demonstrate that neutrophils can be a source of IL-13 and eosinophils and promote M2 polarization [108]. In the transplanted context, neutrophils also can polarize macrophage to M2 towards colony-stimulating factor 1 (CSF1) release, announcing a tolerogenic environment [109]. Marwick et al. (2018) also showed that neutrophils promote anti-inflammatory reprogramming in macrophages by inhibiting the activation of NF-κB [110].

In an inflammatory environment, neutrophils have been shown to impair M2 polarization [27]. This could be mediated by the cytokine TNF-α, which suppresses Arg1 expression [57]. Furthermore, neutrophil extracellular traps (NETs) released by neutrophils can induce polarization of macrophages towards a pro-inflammatory (M1) phenotype and can also create a positive feedback loop by priming monocytes to release CXCL1, enhancing neutrophil recruitment [104].

During the early stages of *Leishmania* infection, neutrophils are among the first cells to be recruited to the site of infection, mediated by chemotactic signals such as CXCL-1, 3, 5, and 8, and LTB4, released by infected cells, including macrophages and dendritic cells, as well as the parasite itself, facilitating their migration towards the *Leishmania*-infected tissue [20,111,112].

After migrating to the site of infection, neutrophils recognize *Leishmania* parasites through pattern recognition receptors (PRRs), and multiple mechanisms are triggered to control the disease [113], including the production of reactive oxygen species (ROS), the release of granule contents, and formation of NETs. ROS, including superoxide anions, hydrogen peroxide, and hypochlorous acid, can directly damage the parasite’s membranes and DNA, destroying them [114,115]. In addition, granule contents such as lytic enzymes, antimicrobial peptides, and proteases can also degrade the parasite direct or indirectly and control infection [112,116]. Finally, NETs, composed of DNA, histones, and granule proteins, form web-like structures that can trap and kill pathogens, including *Leishmania* parasites [117,118].

Besides direct parasite killing, neutrophils modulate the immune response during leishmaniasis by affecting macrophage activation and function [17]. Neutrophils can promote macrophage activation, producing pro-inflammatory cytokines such as TNF-α, which is crucial for controlling *Leishmania* infection [27,119]. Neutrophils also enhance antigen presentation by macrophages to T cells, promoting the development of a Th1 immune response associated with protective immunity against *Leishmania* parasites [112,120]. However, neutrophils can also have immunosuppressive effects during leishmaniasis by producing cytokines such as transforming growth factor-beta (TGF-β) [121], which inhibits macrophage activation and is associated with disease progression.

Considering the significant role of neutrophils in *Leishmania* infection and their ability to modulate macrophage polarization, further investigation into the correlation between these innate cells appears to be a crucial area of study in the context of Leishmaniasis.

### 5.4. T Regulatory Cells

T regulatory cells (Tregs) play a pivotal role in the immune response by attenuating excessive inflammation, preventing autoimmunity, and participating in the resolution of inflammation [122,123,124]. Also, these cells play a protective role in transplanted organs by inducing and maintaining tolerance to foreign antigens [122,123].

Treg cells have been shown to polarize macrophages towards an anti-inflammatory phenotype, enabling them to control the immune response. It has been hypothesized that M2 macrophages and regulatory CD4^+^FOXP3^+^ may be involved in tumor progression by suppressing antitumor immunity [20,125,126]. The IL-10 produced by Tregs suppresses the proliferation of antigen-specific T-cells and decreases the production of type 1 cytokines such as IFN-γ and IL-12. This renders macrophages less responsive to IFN-γ-mediated intracellular killing [127,128]. M1 macrophages produce pro-inflammatory cytokines associated with the expression of inflammatory mediators such as inducible nitric oxide synthase (iNOS) and interleukin-12 (IL-12), which can trigger self-tissue damage. At the same time, Tregs limit the consequences of chronic inflammation and protect the host [129,130].

Some studies unveil the participation of Tregs in the pathophysiology of visceral leishmaniasis [131,132]. Medina-Colorado et al. (2017) observed that during infection with *L. donovani* in hamsters (*Mesocricetus auratus*), there was a significant increase of IL-10 produced mainly by Treg and Th2 in the spleen at 21- and 28-days post-infection [132]. Furthermore, in the later stages of *L. donovani* infection, TGF-β levels increase and bring about a chain of events that hampers NF-kB activation and, consequently, the inflammatory response, thus facilitating the parasite’s survival [133]. In addition, Treg cells secrete IL-13, which stimulates IL-10 production in macrophages [134]. During visceral leishmaniasis, IL-10 plays a crucial role in the immunosuppressive phase of the disease: high levels of this cytokine have been detected in patients with VL. It can lead to a fatal outcome in untreated cases of visceral leishmaniasis [127]. In this way, Tregs may assist in parasite persistence by suppressing macrophage activation and helping the parasite evade the host immune response through the secretion of IL-10 and TGF-β, ultimately resulting in increased host susceptibility and parasite durability [127,135,136].

The dual role of IL-10 and TGF-β in immunity against leishmaniasis is essential for regulating the host’s response [127,135]. On the one hand, these cytokines can increase susceptibility to infection by suppressing proinflammatory cytokines. On the other hand, they can accelerate wound healing and limit inflammation. Moreover, while strong Th1 responses are associated with CL, impaired regulatory T cell (Treg) function causes excessive Th1 reactions and tissue damage [137]. Studies have found that in the skin lesions of CL patients, IL-10^+^ cells are more frequent in CD4^+^CD25^+^ and CD4^+^CD25^−^ populations [130]. Also, the inhibition of IL-10 can promote parasite killing, and the reduced expression of IL-10 mRNA has been associated with healing [138]. Together, these findings suggest that a careful balance of IL-10 and TGF-β is necessary to regulate the host’s response and ultimately determine the outcome of CL [139].

### 5.5. B Cells

B lymphocytes play a crucial role in maintaining the immune system’s function by activating and supporting the survival of T cells, including naïve, activated, and autoreactive T cells [140]. When B cells encounter foreign antigens, they increase the expression of MHC and costimulatory molecules on their surface, promoting T cell proliferation, survival, and differentiation [141,142,143,144]. B cells also produce proinflammatory and anti-inflammatory cytokines, which regulate the immune response and maintain tissue structure [145]. Leishmaniasis is an example of how specific pathogens can manipulate the normal functioning of B cells to facilitate their survival and create long-term infections.

In humans, the strength of the immune response to *Leishmania* infection is variable. It depends on the T cell response, characterized by delayed-type hypersensitivity (DTH) and high levels of IFN-γ. These responses enhance the ability of macrophages to kill the pathogen and control its replication, leading to self-healing cutaneous lesions. However, individuals with a weak DTH response usually have high levels of low-affinity antibodies against *Leishmania*, which fail to control the parasite load and result in evident diffuse cutaneous lesions. Conversely, individuals with a strong DTH and Th1 immune response may develop a severe form called mucocutaneous leishmaniasis (MCL) [146].

In experimental models of cutaneous leishmaniasis (CL), the immune response is inhibited at the infection site by IL-10-secreting CD4^+^ T cells in C57BL/6 (Th1-dominant response) or BALB/c (Th2-dominant response) mouse models [13,147,148]. In several studies, B cells contributed to disease susceptibility [149,150,151]. Depletion or absence of B cells has been linked to enhanced protection against cutaneous leishmaniasis (CL). Continuous treatment with an anti-IgM serum to deplete B cells in newborn BALB/c mice conferred resistance to *L. tropica* and *L. amazonensis* infection. These mice showed a sustained DTH response to leishmanial antigen and could control their cutaneous lesions [152].

Recent studies have demonstrated that Interferon Regulatory Factor 4 (IRF-4) regulates several aspects of B cell function. For example, IRF-4 has been shown to regulate B cell germinal center formation, T follicular helper (Tfh) cell responses, and antibody secretion. Interestingly, it has been demonstrated that mice lacking the interferon regulatory factor 4 (IRF-4) are more susceptible to *L. primary* infection. Furthermore, mature B cells in mice with IRF4 deficiency have an impaired immune response to *L. primary* infection [153,154].

There is increasing evidence in the experimental model of CL indicating that B cell-derived cytokines promote susceptibility to infection. During the early stages of the disease, *L. major* is known to induce IL-10 expression by B cells [155,156]. B-cell derived IL-10, primarily produced by Breg-like cells, plays a crucial role in distorting the immune response towards Th2 cell development and promoting susceptibility to infection with *L. major* LV39 [157].

Visceral leishmaniasis is characterized by hepatosplenomegaly, immunosuppression, and hypergammaglobulinemia [61]. B cells have been demonstrated to be involved in exacerbating the disease, as mice deficient in B cells and infected with *L. donovani* exhibit a high degree of resistance to infection [158]. Recent studies have provided insight into several mechanisms contributing to disease susceptibility, including polyclonal B cell activation, a VL hallmark. This activation induces IL-10 and hypergammaglobulinemia, producing low-affinity antibodies against the parasite [159,160,161].

Similarly to CL, B cells are also involved in producing IL-10 and contributing to disease susceptibility during VL [159]. However, the production of IL-10 during VL is mainly attributed to MZB cells, which depend on myeloid differentiation primary response 88 (MyD88) and endosomal TLR signaling pathways [159,161].

B cells have also been found to suppress T cell functions via IL-10 in the canine model of VL and human VL patients [155,162]. It was demonstrated that the secretion of IL-10 depended on the activation of spleen tyrosine kinase (Syk), phosphatidylinositol-3 kinase, and P38 mitogen-activated protein kinase (P38) [162]. B cells express multiple cytokines throughout *L. donovani* infection, including IL-1α and β, and type I interferon (IFN-I), indicating that IL-10 is not the only immunomodulatory cytokine produced by B cells during VL.

Increased disease susceptibility has been associated with IgM and complement activation [160]. IgG immune complexes can also enhance IL-10 production in macrophages, which promotes disease [163]. Additionally, during active VL, polyclonal B cell activation leads to the production of autoreactive antibodies [164]. Despite the role of B cells in VL, it is noteworthy that high-affinity, *Leishmania*-specific antibodies are not typically produced during chronic infection. This may be due to a significant reduction in T follicular helper cells during this condition stage [165]. In another infectious disease, tuberculosis, Bernard et al., 2018, suggest a correlation between the innate production of type I IFN by B cells and the altered polarization of lung macrophages during Mtb infection [166].

## 6. Final Considerations

**Figure 3:**
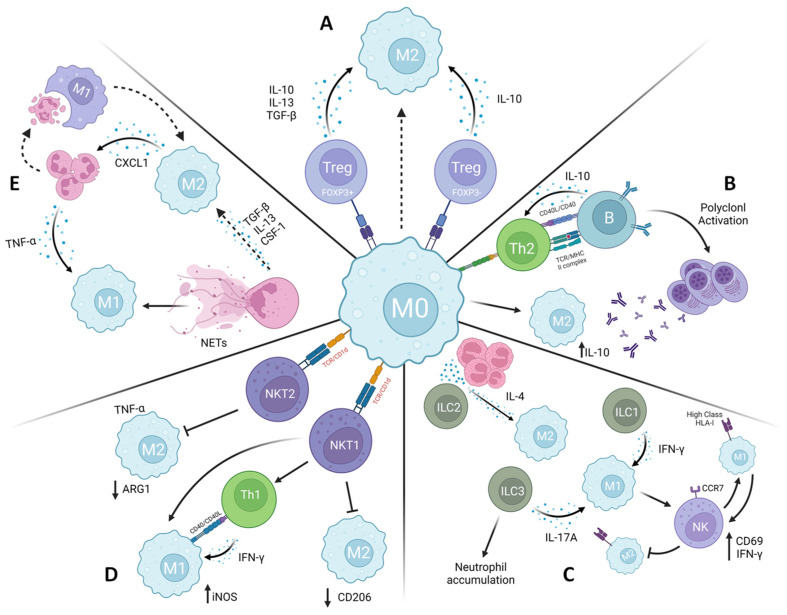
Crosstalk between immune cells in macrophage polarization. Macrophage polarization could be modulated by different cells in a microenvironment-dependent manner. (**A**) Regulatory T cells (Treg) can induce M2 phenotype by releasing cytokines (e.g., IL-10, IL-13, and TGF-β). (**B**) B cells can induce macrophage towards also to an M2 phenotype by releasing IL-10 and supporting Th2 polarization, a known cell to induce M2. (**C**) Innate lymphoid cells (ILCs) can promote M1 and M2 polarization, mostly by releasing cytokines. ILC2 releases M2 phenotype inductor cytokines (e.g., IL-4), while ILC1 and ILC3 induce M1 phenotype by secreting IFN-γ and IL-17A, respectively. M1 macrophage can activate NK cells, which can lysis M2 macrophages, but not M1 macrophages. (**D**) NKT1 cells can support Th1 polarization and, thus, M1 phenotype while can reduce M2 CD206+ frequency. M2 polarization can be inhibited by NKT2 cells. (**E**) In specific microenvironmental conditions, M2 macrophages can attract neutrophils by releasing CXCL-1. In turn, neutrophils have a dual role in macrophage phenotype. Through TNF-α and neutrophil extracellular traps release, M1 polarization prevails. In contrast, releasing other cytokines (i.e., CSF-1, IL-13, and TGF-β) and efferocytosis (i.e., macrophage phagocytosis of apoptotic neutrophils) lead to M2 polarization. Created with BioRender “https://app.biorender.com/ (accessed 21 April 2023)”.

The collaboration among immune cells is crucial for upholding the integrity and optimal functioning of the immune system. Moreover, this coordinated immune response raises the possibility of developing a series of immunotherapeutic approaches [167]. In particular, the intricate interactions between macrophages and other immune cells (Figure 3) reveal a vast potential for investigating the role of M1/M2 macrophage polarization in leishmaniasis and how it may be controlled not only by Th1/Th2 cell responses.

## Data Availability

Not applicable.
